# Fourth Annual Meeting of the American Dental Convention

**Published:** 1858-09

**Authors:** 


### FOURTH ANNUAL MEETING OF THE AMERICAN DENTAL CONVENTION.

("Reported expressly for the" Dental Register. By our own Reporter.)

### GENERAL REMARKS AND DISCUSSIONS.

The fourth annual meeting of the AMERICAN DENTAL CONVENTION, was held in Cincinnati, the 3d, 4th, 5th and 6th days of August, 1858.

In accordance with the report of the Executive Committee, the address of the President being in order, Dr. James Taylor of Cincinnati, the acting President, stated that, by the dispensations of Providence, he had not been able to prepare a written address, as he fully intended to do. He, however, proceeded to deliver an extempore address, which was listened to with much interest, and which he has not been able to write out for this number.

The election of officers occupying the remainder of the morning session, the Convention adjourned to 3 o'clock, P. M.

FIRST DAY—AFTERNOON SESSION.

The retiring President appointed Dr. Allport of Illinois, and Dr. Chase of Georgia, to conduct the President elect to the chair. He was introduced in a few appropriate remarks by Dr. A., and, on taking the chair, made a brief and appropriate address, of which the following is but a mere outline :

He thanked the Convention for the honor just conferred—he regarded it highly as an honor—bestowed on himself individually, but felt much more gratification in viewing it as a compliment to St. Louis, of which city he was a representative. He would make a few remarks on this Convention. It was founded by the great and good members of our glorious profession ; and one of its leading objects was to demonstrate the advantages to be derived from local associations. In this convention each member is responsible for his own moral and professional standing—each takes the responsibility of his his own remarks ; but it is not so in local associations. There the society is responsible for the moral character and professional attainments of its members. He alluded feelingly to the late sad bereavement of the retiring President who had just buried his wife.

He remarked that it would be easy to preside, if all would co-operate with him. We must consider ourselves one. I want your experience—you want mine. We can perceive each other's weak points, and give support where needed ; and we can perceive each other's strong points, and take advantage accordingly. To the beginner—the rising student —he would say, that as age advanced, and experience extended, he would find himself still a student. He advised all to be, in the strictest sense, honest—*honest.* Even if adopted from policy, honesty is profitable. The physician's patients and mistakes are often buried together ; our patients live to complain, and to exhibit the evidences of our lack of skill, or want of honesty. The best operators may fail, but still he thought he could tell, by inspection, when an operation had been *honestly* performed. A new student, he said, was apt to think himself about the best dentist in existance; but after a time, he recovered from such fancies.

Essays being called for and none presented, the President announced as now in order, discussions on the general topics reported by the Executive Committee. Before discussion began, it was resolved that on each topic, written remarks be first in order. (For subjects of debate, see Minutes in this Number.)

I. *The best means of securing good Teeth.*

Written remarks being called for, Prof. J. Richardson read a paper, which was followed by the reading of another by Dr. W. B. Ingersoll, of Ohio. Both of these papers may be found in the original department of the present Number of the Register.

Dr. W. H. ATKINSON remarked that this was a common-place matter, and should be better understood. He said the best means of producing a healthy person, are the best means of securing good teeth. Healthy individuals are likely to be produced only from healthy children, begotten by healthy parents, under proper circumstances. But, as it is mostly impracticable to begin so far back, we must attend to minute matters as they present themselves. The primary teeth should be carefully preserved, that the permanent may be healthy. Many of the profession, he feared, were faulty in this, and parents are extremely careless in the matter. The first permanent molars were often lost, by this want of care, as they were mistaken for primary organs, and were left to their fate. He advocated filling the primary teeth before the pulp is exposed. His experience was that when the pulp of the deciduous organ is lost, its roots are not normally absorbed, to make way for the permanent tooth.

Dr. BERRY said the best way to avoid sickness, is to keep well. But in securing good teeth, many things must be attended to. We wanted good stock to begin with. The parents should be healthy—and the grand parents—as hereditary pre-dispositions often jump a generation. To be safe, the dust from which we are created, must be right. It is important, he said, to inquire if, in ordinary food, we get enough of phosphate of lime, to properly develope the teeth and bones. Unfortunately, fine flour contained but little of it. It is mostly given to the horses and hogs, in the form of bran. If we would diet as Adam and Eve did, we might do better. Beef and pork, he supposed, contain but little of it. He preferred the bill of fare published in Genesis i. 29. He would make beef-steaks of pears, plums, peaches, etc., and the bread, he would make from the grain taken "just so."

Dr. PERINE said, that dental disease is not new; but it had probably increased, though he knew not why. He had tested the medicinal use of phosphate of lime in his own family, and found it satisfactory.

Dr. ROGERS, of Utica, said it was not worth while to mention sound teeth as an argument against improper marriages. Stronger arguments were constantly brought to bear, yet they had no influence as long as people married according to their tastes and not their judgments. He did not believe that even well formed teeth would ordinarily last through life. He illustrated this idea by the fact that the teeth of the English are good when they come to this country, but soon degenerate, like those of the natives. It would not do to attribute bad teeth to any one cause. He had theories, but had little confidence in them.

Dr. WRIGHT said it was difficult to determine what is practicable. He had practiced twenty years and was still at a loss. People will marry "in spite of their teeth." If he had all the match-making for two or three centuries, he thought he could raise a race with good teeth. He believed bad teeth are often inherited. He has lost the same number of teeth as his mother, and the positions of those lost, exactly correspond. He could refer to a number of similar cases. This tendency, he thought, could not be prevented. He was at a loss what to say when a parent inquired what a child should eat. He did not believe that sugar injures the teeth. He did not believe that decay of the teeth always begins externally. He thought it was as frequently internal in its origin as external.

Dr. Watt remarked that though he differed with some who had preceded him, yet he was much gratified with the remarks offered. It was important, he said, to notice two general points. The teeth should be properly developed; and they should be properly protected during development. He did not believe in internal decay of the teeth, and thought it would not do to lose sight of the chemical view of the question. No tooth is so badly developed that it will fall to pieces spontaneously, and none so well that it can successfully resist the action of chemical agents, liable to be brought in contact with it. A well organized, solid tooth, however, would much better resist chemical action, than one otherwise constituted. It was important, then, to pay attention both to mother and child; and this he considered a *practical* question, and practiced accordingly.

SECOND DAY—MORNING SESSION.

The first subject, the best means of securing good teeth, being continued, Dr. Frank Fuller remarked, that it was a difficult and interesting question. If we do what we can for the health of the mother, that is all we can accomplish in this direction. He could scarcely understand what was claimed for the phosphate of lime, and would hardly administer it in a state of health, even if the mother's teeth were defective. He recommended a healthy regimen. Bad teeth, he said, were not usually found among the active, laboring classes, but with those who were confined to bad air, and led artificial modes of life. But, when the period of eruption arrives, much may be done. The doctrine of internal decay, as suggested yesterday, he considered altogether unsound. The diet, he said, was very important. The Irish, whose diet is principally potatoes, and who lack animal food, have very brittle teeth. He thought this evidenced a lack of earthy matter in the teeth. An intelligent German, in conversation with him, remarked that, his teeth being good when he came to America, but decaying soon after, he imputed the defect of the teeth in this country to the quality of the food used. In Germany he used hard food, which kept the teeth clean, and they remained sound; here he used soft food, and his teeth decayed. He, (Dr. F.), thought the climate had less to do with the decay of the teeth, than the style of living.

Dr. Atkinson inquired if Dr. F. thought that the brittleness found in the teeth of the Irish, was produced by defect of earthy matter.

Dr. Fuller said that was his opinion.

Dr. Atkinson thought it arose from a defect of gelatine, and a relative excess of earthy material.

Dr. Roberts remarked, that much had been said of constitutional influence, that bad teeth are hereditary, etc. The doctrine may be true to some extent, but not so far as some might suppose. A pure African has a good constitution, but by mixture with other races, it degenerates. The population of the United States is compesed of a mixture of races. These all differ in physiological characteristics. A mixture of these races causes a disproportion in the form and size of the teeth and jaws. These thoughts, however, he said were theoretical, not practical. He would not tell a lady she had married the wrong man. He would take as good care of the child's teeth as possible. Cleanliness, not only of the mouth, but of the whole body, was important. He did not believe in internal decay, as suggested yesterday. The enamel being placed on the outside, indicates that the danger is from external sources. It extends only to the gum, then another substance acts as a protector, and who, he inquired, ever heard of caries attacking the roots of the teeth?

Dr. Tefft, of Vermont, stated, that through his brother, a foreign missionary, he had some knowledge of the teeth of the Africans, in their native country. In their barbarous state their teeth are good; but, when introduced to civilized life, they decay. He remarked, that on the east side of Lake Champlain, the teeth are attacked with "black decay," while in western New York and Michigan, the white is the prevailing type.

Dr. Rehwinkle said, that if the health were perfect, there would be no decay of the teeth. Uncivilized nations live more in accordance with nature. Decay of the teeth was evidently a chemical decomposition. Cleanliness was very important. He thought the use of lime-water and prepared chalk, as prescribed by Dr. White of Philadelphia, a good suggestion. The constitution of the child might be reached with advantage, sometimes, through that of the mother. He thought an excess of earthy matter rendered the teeth brittle. In Ireland, he said, inflammation of the gums is a prevalent disease. The constitution was often injured by malaria; and the fluids, being thus depraved, act injuriously on the teeth. In the malarious districts of France and Germany, he said, the teeth are not good.

Dr. Kelsey, of Mt. Vernon, remarked, that we can not control marriage,—we must, therefore, take children as we find them. The health of the mother should be carefully watched, and she should have a healthy diet; but, after all, cleanliness is the great thing. He advocated filling the primary teeth, and maintained the chemical theory of decay. Both sets of teeth should be carefully watched, and the primary should not be prematurely extracted. He would not go into the mists of the internal decay question. It belonged to the physiologist rather than the dentist.

Dr. Lyman, thought the whole subject belonged specially to the dentist. If physiology does not belong to dentistry, he would inquire, what does belong to it?

Dr. STONE said that the predisposition to decay is evidently hereditary; but we could counteract the tendency. The constitution could be materially modified. He recommended friction of the gums, from the earliest infancy. His own teeth are defective, and his wife's too; yet his children, he said, have all good teeth. He referred to a case—a physician's daughter. Her teeth were sound at twelve years of age. At thirteen, there were twelve decays. She drank no water—used milk almost exclusively. Her saliva was ropy, or tenacious. He directed her to drink water, use but little salt, *no milk*, no coffee. The ropy saliva disappeared; and the tendency to decay was corrected.

(Were the twelve decays all of the same charcter? And if so, what class did they belong to? Will Dr. S. please write to us?—EDS. REGISTER.)

Dr. ROBERTS, advocated careful friction, or washing the gums in infancy. It usually prevented sore mouth, and promoted healthy dentition.

Dr. ASAY, considered cleanliness the important means. Early attention to the gums of infants, kept the mouth healthy, and rendered dentition easier. He recommended the use of a soft napkin, for washing and rubbing the gums. The Irish have usually white decay, after they come to this country. He thought a large per cent. of the miseries of life, is due to neglected teeth.

Dr. BRANCH said, that to secure good teeth, we must secure a healthy constitution. Whatever is deficient in the system, should be appropriately supplied; and the temporary teeth should be retained as long as possible.

Dr. CHANDLER said, that the subject is beset with difficulties, but this should only impel us onward. The diet of both mother and child, he thought very important. He remarked, that, as a rule, in the South, house-servants have bad teeth, and field-hands good ones.

Dr. TAYLOR said, that science had already furnished us with a few leading facts. We know that there are certain elements in the teeth, and that certain articles of diet furnish these elements. Then, different temperaments appropriate and use these elements according to their relative forces. The sanguine temperament, furnished with the same materials, would develope better teeth than the lymphatic. The temperament, however, might change, and the character of the teeth change with it. So far as the dental organs are concerned, he concluded there was nothing deleterious in our climate. The aborigines had good teeth, *even on the salt-water coast.* The constitution of the mother influenced that of the child. The blood of a child from a diseased mother, is impaired. An unhealthy mother was not, he said, a suitable or successful nurse. And here a practical question arises. If we can not impart a normal quality to the mother's milk, we can, at least, recommend that the child be supplied with a healthy nurse. He regarded the phosphate of lime, as an important remedial agent, in defects of the osseous tissues. It should be administered, in many cases, where it is deficient in the system. He spoke of its relative quantities in bran and flour, and thought the system would be better nourished by the use of the entire grain. He remarked that the constitution could often be braced up, till the structure of the teeth so improved that they could better resist disease. He remarked, that not only the character of the constitution, but even the state of the health could be ascertained by careful inspection of the teeth.

Dr. Rogers of Cincinnati, regarded this as a most important question, and said that whatever is done, practically, to secure good teeth, must be accomplished in early life. Decay of the teeth resulted from defective enamel, or from vitiated fluids. As the enamel is all-important, our attention should be enlisted during the period of its formation; for it is not changed much after it is formed. The deleterious influence of malaria here, he observed, was similar to its influence in Germany, as described by Dr. Rehwinkle. What can be done to counteract the influence of malaria, he regarded as an important question.

Dr. MURRAY thought it was important to avoid mechanical injuries to the teeth. Even in early life, he said, the dental organs are used as a substitute for hammers, vices, pincers, etc. He said he was alarmed to hear that Irish teeth were bad. Being once in that country, and standing in need of teeth, he secured a deciduous set, as an experiment, which lasted him a few years. Being about to leave, and being told that the teeth furnished in that country were good, he secured a *second* set, which had lasted him till the present time. He thought, that by avoiding mechanical injury, and by cleanliness, even Irish teeth would last a good while.

Dr. DECAMP remarked, that the question seems to have as many horn's as Daniel's goat. An important step would be to make every mother a physiologist. The teeth, he said, are mainly developed before we have anything to do with them. It is important, he thought, to modify and improve public opinion. To carry out every process successfully, nature must have the material to work on, the tools to work with, and the ability to accomplish the work undertaken. It is our duty to inform the public on this matter ; and if physicians will coöperate with, future generations will bless us.

Dr. MCKELLOPS said, that the good teeth of foreigners seemed to be a favorite topic. He thought there was probably less difference between the dental organs of the old world and the new, than was generally supposed. In Europe, dentists are not so generally distributed as here ; and, consequently, the teeth are not so closely observed. If chemistry causes the decay of the teeth, he thought it could also furnish a remedy. Why not, said he, have a chemical wash ? He wished to know when gentlemen consider it proper to fill deciduous teeth ? He inquired if there was not danger of injuring the permanent organ.

Dr. DUNHAM said, this subject belonged more appropriately to the journals, than to a popular meeting. He said most diseases are hereditary, and were less dependent on local causes, than was generally supposed. His own teeth, he said, are good, though he is a dyspeptic. His brothers and sisters have bad teeth, though they are healthy.

Dr. Wright said, that to secure a healthy permanent denture, it was necessary to retain the deciduous teeth as long as possible. He would fill them when practicable. The objection to premature extraction is, that the jaw contracts; yet, if a deciduous tooth be so diseased as to affect the constitution, he would remove it.

Dr. Allen said we must be careful to avoid extremes. He was in favor of saving the temporary teeth by filling, when it can be done; but there was danger of injury to the permanent organs, by undue pressure. He had met with such results in his own practice.

Dr. Taft said, the remark had been made, that it was proper to retain the deciduous teeth *as long as possible.* When they are not shed at the proper time, making due allowance for constitutional differences, and if the permanent teeth do not follow them directly, he would remove them. They should be retained till the right time; and should not be permitted to become diseased. However, it was often necessary to remove them. When decayed, and he thinks it practicable, he fills them. The pressure *need* be no greater, he thought, than in mastication. Within a year or eighteen months of the period of shedding, he would not consider it proper to fill them; but, three or four years previous to shedding, the attachment is so firm that the pressure need not be dangerous. He considered it a very delicate matter to destroy the deciduous pulp. The parts around a tooth were less likely to remain healthy after the death of the pulp, especially around deciduous teeth. It was important to disscriminate. In some constitutions he would not attempt the operation, Indeed, he would only occasionally destroy a deciduous pulp.

II. *The best means of preserving the teeth.*

Dr. WATT said, that good teeth being "secured," the danger to them arises from the presence of substances capable of acting chemically upon them. These substances are to be found among the acids. It is true that mechanical injuries, and other causes, do very much facilitate the action of these agents; yet fracture of the enamel would be a thing of small moment, were none of these agents brought in contact with the tooth. The time was coming, and he believed it was nearly here, when the intelligent dentist would be able to tell precisely what agent produced any given case of decay. When this attainment is made, we can treat the mouth in a rational manner. It was evident, he thought, that many acids, capable of acting on the teeth, have no direct influence in producing dental decay. It was not the brief, accidental presence of acids in the mouth that causes decay, as we ordinarily see it, but the slow and prolonged action of those agents, in a diluted state. Those acids which produce dental decay, he thought, were brought in contact with the teeth by *secretion*, as when the functions of a secreting organ are depraved,—by *excretion*, as when the elements which compose them are in excess in the system,—by *fermentation* or *putrefaction*, as when particles of food are left to decompose in the mouth. All the four recognized varieties of dental caries can be produced, or at least imitated, artificially, by the use of acids. For example, "white decay" may be readily produced by nitric acid; and as yet he knew of nothing else capable of producing it. If a portion of muscular fibre be left to putrify, between the teeth, ammonia is invariably formed; but, in the presence of ammonia and atmospheric air, nitric acid is always formed. White decay on approximal surfaces, was thus easily accounted for. Now, constant attention to cleanliness, would remove the particles of food, and also the secreted and excreted acids; but something more, he said, was desirable. It would be well to prevent the formation of the acids. He illustrated this point by allusion to a little dog, chasing away the locomotive. His watchfulness was thus far successful, as it had done no damage; but if the little fellow could tear up the track and thus prevent its coming, the course would be more philosophical and satisfactory. (The President, by courtesy, or *oversight*, indulged Dr. W. far beyond the allotted ten minutes.—Rep.)

Dr. Berry stated, that he had been informed, on good authority, that the Japanese have usually, good teeth. He exhibited a tooth powder used by that people, which, he said, might be interesting to the curious. A powder, similar in appearance and physical properties, might be made from prepared chalk, pumice stone and rose red.

Dr. Van Emon inquired of Dr. Watt, what influence galvanism had on the destruction of the teeth.

Dr. Watt said, it had no *direct* influence. It could only act on the teeth, by forming or eliminating compounds, capable of exerting an influence on them.

Dr. Taft said, that to preserve good teeth, the secretions of the mouth should be kept normal. If they become abnormal, it was important to know how to change them. To remain healty, a tooth must be exercised. A tooth without an antagonist, we all knew, was likely to become unhealthy. The use of hard, firm food, has a tendency to preserve the teeth. Dogs, he said, have no tartar on their teeth. The character of the food is not sufficiently cared for. The state of the buccal secretions, he said, had much to do with the question of cleanliness. They were sometimes so adhesive, that they attached themselves to the teeth with considerable tenacity, and were not removed except by careful brushing.

Dr. Stone, requested Dr. Taylor to give his views of the use of sugar, salts, candies, etc.

Dr. Taylor said, he did not consider candy very good diet. Corn bread, he thought, was undoubtedly, better. Sugar, he thought, had no bad effect on the teeth—was a useful article of food.

III. *The treatment of exposed nerves.*

SECOND DAY—AFTERNOON SESSION.

Dr. Atkinson said, that when the nerve was accidentally exposed in operating, he applied creosote on a pledget of cotton, and pressed it, with a blunt smooth instrument, on the exposed point, continuing the pressure, in many instances, for a minute or two. He then placed a thin horn cap over the exposed part, pressed it to its place with cotton on a blunt instrument,—the pressure of the atmosphere would hold it in its position. Then he would fill the cavity as usual. If the pulp was exposed when the patient first came, but not inflamed, he would pursue the same course. If inflamed, he would, if practicable, reduce the inflammation, and proceed as before. Many substances, he said, would do for caps as well as horn, but this was so easily prepared, that he preferred it. By pounding, or hammering the horn, it is divided into thin lamina, very convenient for use. Under this treatment, he said, the nerve would often be protected by a deposit of cementum. He referred to a case of exposed nerve, in which the tooth was filled, February 23d, and on the 22d of March following, the filling was removed to ascertain the condition of the tooth. The nerve was covered by cementum.

Dr. Branch said, that he filled such cases without applying creosote, or any thing else. He had seen inflammation result from the use of creosote.

Dr. Asay feared he was behind the age. Three fourths of the cases he capped, failed in less than six months. They generally inflamed. His usual practice was to destroy the nerve, and fill the fangs.

Dr. Tefft said, that his method was, to destroy the nerve with arsenic, tannin, and creosote, and he did not fail more than one time in fifty.

Dr. Wright said, that when there is slight exposure, he touches the exposed point with nitric acid, and filled without capping, and in fifteen years had not lost a case. The nitric acid, he said, blackens the tooth. He had not been very successful in taking out nerves.

Dr. W. B. INGERSOLL reported a case in which he performed what was, at the time, known as "the Hullihen operation." The tooth was a lateral incisor, with exposed nerve. The operation was performed five years ago, and the tooth is still healthy. He had failed in other cases. He had tried capping, but didn't like it. He now removes the nerve, unless the tooth can be filled without capping.

Dr. STONE said, his practice was to remove as much of the decay as he could, without causing severe pain, and then extirpate. He had quit capping, as he could not get teeth, treated in this way, to last over two years. He removes the nerve with an instrument, wipes out the blood, and fills in a few minutes.

Dr. PERINE said, he once extirpated all nerves, but, on receiving suggestions from Dr. Atkinson, he had changed his mode. He thought it important to preserve the vitality of the tooth.

Dr. JOHNSON, of Indianapolis, said he applied chloride of zinc directly to the exposed nerve, repeating the application if necessary. The pain was severe. Its application, he thought, was more successful with young patients than with old. He had used the chloride in this way for two years, and had seen no discoloration of the teeth.

Dr. FULLER said, he could not get along without the chloride of zinc. It is a good antiseptic, as well as escharotic. He thought it important to fill the nerve cavity. He destroyed nerves with escharotics. He formerly used arsenic and morphine,—now he omits the morphine, and finds that less pain is caused by the escharotic action. He now uses chloride of zinc, instead of creosote. In using the latter, the nerve sometimes dies, and the tooth becomes discolored.

Dr. DUNHAM said, he had capped and failed; and he had succeeded with various caps. The tooth could often be saved, after the nerve is destroyed, without filling the canal; but he usually fills it. Plaster of Paris, he thought, formed the best cap.

Dr. ALLPORT said, he was not very successful in treating exposed nerves. If it was inflamed, he cut it out; if not, he tried to save it. He uses arsenic occasionally.

Dr. ROGERS of Utica, thought, that if a nerve was seriously wounded, it would certainly die.

Dr. REHWINKLE thought, when a nerve was exposed, its case was almost hopeless; if suppuration had begun, it was entirely so. If inflamed, and but lately exposed, he would use astringents instead of escharotics, and insert a temporary filling. He reported successful cases treated in this way. He said (to Dr. Atkinson) that osteo-dentine is sometimes formed under such circumstances.

Dr. BRANCH said, that when it was necessary to extirpate the nerve, he would apply cold water, then ice-water, then a freezing mixture, and remove the nerve with an instrument. He uses no morphine in arsenical mixtures for destroying the nerves, and the result is, there is no pain. He uses a bristle instead of a broach. In the use of caps, he succeeds in three out of four causes. He had seen exposed nerves protected by a deposit of cementum.

Dr. WRIGHT reported a case in which the pulp of a molar was wounded and bled profusely. The tooth was filled with tin, a pledget of cotton being left in the cavity. Twenty-four years afterward, he removed the filling, the cotton was in a good state of preservation, osteo-dentine was deposited over the nerve, and the tooth was sensitive, and evidently alive. He refilled it successfully.

Dr. ROGERS of Utica, said, he would prefer to believe that Dr. W. had been misinformed respecting the condition of the tooth at the time it was filled. He would not say that the nerve always died in such cases, but he believed it did.

Dr. BERRY said, that if he wounded the nerve, he expected it to die. He thought, however, he had seen one successful case in his practice. He extirpates the nerve with an instrument, and said that in the South they had the *nerve* to stand it.

Dr. PALMER said, that the pulp may be exposed at minute points, and the operator not be aware of it; and if a little blood be left in the cavity, suppuration, and consequent pressure, would result. In such cases he applied creosote, with a burnisher, over the entire surface. If the exposure be considerable, he would moisten a pledget of cotton with creosote, and press it firmly in the cavity, for some time, then fill up tightly with cotton awhile, and afterward fill with gold, without a cap. When the nerve is extirpated, he fills the fangs. If the nerve is freely exposed, he applies a muslin cap moistened with creosote, and fills over it. He is opposed to the use of arsenic, though, he said, he was among the first to use it. He thought morphine combined with it, increases the pain. He now uses cobalt, which is milder.

Dr. ROBERTS said, he removes the nerve, when practicable, with an instrument. The operation is not as severe as patients expect it to be. When a front tooth is aching, and the nerve is exposed, he removes it with an instrument, to relieve the tooth-ache, and then fills immediately. The experiment of saving exposed nerves he thought well worth a trial.

Dr. TAYLOR said, his practice was somewhat modified. He was in the habit of capping, more or less. He discriminated more carefully than formerly, but was in favor of capping in appropriate cases. He said it was important to know whether the decay reaches the pulp or not, before we cut down to it. If the tooth is sensitive around the border of the cavity, the nerve, he said, is not exposed. If a nerve was wounded, he formerly destroyed it; but he began to inquire why a wound of the pulp membrane should give more difficulty than cutting off the nerve and vessels in the canal. Resoning on this, he began to cap and fill, even if the pulp were wounded, and was usually successful in favorable constitutions. If the constitution were bad, and the mouth diseased, he would not try it. He does not use morphine and arsenic to destroy nerves—uses creosote and cobalt. He inquired what should be done with a nerve that arsenic would not kill.

Dr. CHASE used creosote and arsenic, applying a lead cap over it, to prevent pressure on the nerve. The result was, no pain from the action of the arsenic.

Dr. ALLEN said, he used creosote and arsenic, and there was no pain, unless it was caused by pressure.

Dr. OSMOND said, if the pulp be wounded, there will be hemorrhage and effusion of lymph, and, in good constitutions, a deposit of osteo-dentine. In scrofulous cases, he recommended the use of hypo-phosphate of soda and lime, to promote the formation of this deposit. His favorite cap was prepared by folding foil to the proper thickness, and varnishing it with gutta percha dissolved in chloroform.

Dr. ULREY inquired, if those who use arsenic ever witness bad effects from it. He thought it important to report the failures and mishaps, as well as the successes.

THIRD DAY—MORNING SESSION.

Exposed nerves continued.

Dr. FORBES, the President, stated, that if the nerve was freshly exposed and uninjured, he would let it alone, and fill over it. If wounded, he would apply tannin and glycerine, and fill. When he can not save the nerve alive, he extirpates it with an instrument, if he is smart enough. If the patient proved too smart for him, he destroyed it with an escharotic. He believed that was all.

Dr. KELSEY said, his patients were too "smart" to have their nerves extirpated with an instrument. When the pulp was wounded, he applied chloride of zinc and tannin, for the purpose of saving it. It was important to avoid pressure by too great expansion of the nerve. He said there were practical difficulties in extirpation by instruments, such as divergent roots, angles, etc. He was in the habit of using crude arsenic and chloroform, to destroy the vitality of the pulp.

Dr. Rogers of Utica, suggested, that the vitality of the tooth was not lost by the death of the pulp, and that, therefore, these remarks which conveyed that impression, were unguarded or incorrect. In using arsenic to destroy the pulp, he advised coating the surface of the cavity with wax, then displacing a portion of it over the nerve, and applying the escharotic to that point. He thought the tooth was less likely to be discolored. Over the arsenic he applies plaster of Paris, when the cavity is accessible, or in such position that he can drop it in. He believed that to fill over the nerve when it is nearly exposed, it will generally die.

Dr. Taft said, that in all that had been said on this topic, we had heard little or nothing in reference to the character of the constitution. As far as described, we would infer that all cases are alike. Now, he said, if we meet with an exposed nerve, in a constitution of low vitality, it is useless to try to save it. But, when the constitution is good, we can save a majority of the cases. In many favorable cases, he said, the nerve would be protected by a deposit of osteo-dentine. He had frequently found this to be the case, on removing fillings, to ascertain the condition of the teeth. For caps, he was in the habit of using oil-silk, horn, gutta percha, and similar non-conductors. After a tooth was thus filled, however, it might be lost by constitutional disturbance. If the exposure was large, and the pulp healthy, he would generally proceed as above. If inflamed he would protect it from irritating substances, and apply constitutional treatment; or, if necessary, he would resort both to local and general treatment. The vitality of a tooth, he said, was dependent both on the internal and external membrane. The vitality of the crown was lost by the destruction of the pulp. In reply to a question, he said, that when the exposure is slight, and the constitution good, nineteen in twenty cases might be saved. He based the statement on actual examination of cases, in which he had removed the fillings to ascertain the state of affairs.

IV. *Mechanical Dentistry.*

Dr. FRANK FULLER, by request, described his mode of inserting partial sets, single teeth, etc., on suction plates. He cuts out the central part of the plate after stamping partially, then, after having made it fit his cast and plaster model perfectly, he stamps another portion of plate over it, to form the chamber, and solders it. The chamber has thus a definite border, and is, at its margins, only the depth of the thickness of the first plate. (As well as we could understand him, it is made in the same manner as the Cleaveland chamber, except that no space is left between the plates.—REP.) The square edge he considered a decided advantage. A chamber otherwise formed, he thought tended to draw an excess of the membrane into it, consequently it soon became filled, and there was, of course, no vacuum. He remarked that the same principle had been applied to breast pumps, and the result was, equal suction with much less pain. In forming the chamber, he thought it was important that it be not placed too far forward. When it is, the plate glides forward as it adheres.

Dr. MORGAN said, that his mode was to form the chamber, by striking up the plate over a lead pattern. His experience differed from that of Dr. F. in regard to square-bordered chambers. He preferred a chamber formed by swaging, to the Cleaveland chamber, or even to the modification of it practiced by Dr. F. "When the plane of the chamber looks forward," he thought the plate would run forward.

Dr. CHASE said, he used the Cleaveland chamber seven or eight years, but had abandoned it. It produced irritation, and caused an excresence on the membrane that was both unsightly and annoying. He inserts nearly all his partial jobs on suction plates. In swaging a chamber, he made its borders as square and definite as practicable. In soldering two plates together, he thought there was great danger of warping, To prevent the plate running forward, he let a little spur of the plate, having its border thickened, rest against a tooth. He had found this altogether satisfactory.

Dr. Murray said, he had seen a piece of work which had been worn some time, which gave him a prejudice against "cut out" chambers. The cavity was very deep, and was filled by an enormous fungous growth. After two weeks' treatment, with the plate out, the tumor was still quite prominent. He said he could stamp a chamber with borders "square enough."

Dr. Allen having been absent at the commencement of this topic, now read a paper, reviewing the subject at considerable length. (This paper is in the original department of the present number of the Register.—Rep.)

Dr. Roberts described at length his manipulations in making continuous gum work, illustrating each part of the process with specimens. He spoke also of the general properties of platinum, and of the difficulty in preparing its scraps for use, (We are unable to do even partial justice to Dr. R. on this subject; but as he has promised to write out his method for publication, we can afford to wait, hoping that he will speak for himself in the next issue of the Register. —Rep.)

Dr. Vandervert said, that as to the difficulty of uniting platinum scraps, he could weld them together with no other appliances than a blow-pipe, piece of charcoal, a hammer and an anvil. Dr. V. went down to the laboratory of the College with Dr. Roberts, welded two pieces together, and returned. The welded specimen was passed round by Dr. R., and Dr. V. was requested to describe minutely, his manipulation. He said, if he wished to unite a number of scraps, he rolled out the largest one quite thin, and enveloped the others in it, then heated the mass to a high heat, placed it quickly on an anvil and gave it a blow with a heavy hammer, then reheated and gave it another blow, and so on, changing its position each time. In a short time, he said, the whole mass would be thoroughly welded, and could be rolled into plate as desired.

On motion, this topic was laid aside for the time, and the subject of filling teeth was resumed. It was suggested that each member take a special cavity, and describe his mode of filling it.

Dr. ATKINSON described his method of filling the second left upper molar, the cavity being in the posterior approximal surface. He said, that if he could find a lawful excuse for extracting the third molar, he would like to have it out. But if his conscience would not let him remove it, then he would separate freely, so as to have ready access to the cavity. After removing the decay, he would form grooves or pits, as retaining points, insert and condense his gold as usual, finishing near the middle of the cavity.

Dr. TAYLOR inquired how Dr. A. would manipulate if the patient had long hair all around his mouth.

Dr. ATKINSON said, he would shield that below the mouth with the napkin,—that above he would stick together slightly with gum mucilage, and lay it to one side. He said he often used the mallet in condensing the gold, and maintained that less violence was necessary by this mode than by any other. It was important, he said, to go over the gold but once, with the condensing instrument, as otherwise, the next piece would not adhere so readily. When the gold is sufficiently condensed, the mallet rebounds from the instrument.

Dr. ALLPORT selected an anterior approximal cavity in the left lateral incisor. He would separate by wedging, if practicable Even if the patient were not young, he would resort to this mode, if other circumstances were favorable. If necessary, however, he would file—he would obtain space sufficient to enable him to reach every point of the cavity. For the cavity under consideration, he would use very fine pointed pluggers. He would insert a piece of wood between the necks of the teeth, to keep the upper portion of the cavity dry. The great danger of defect was next the gum; and, to obviate this, he would insert a large cylinder at this point, and press it firmly into place, with a flat, smooth instrument, leaving a portion of the cylinder projecting from the cavity. Then he would add, successively, soft cylinders, till but a small portion of the cavity, next the cutting edge, is left. This he would fill with crystal gold, or adhesive foil, and but little pressure would be required to render it efficient. He did not say that this would make a *perfectly solid* filling. He did not believe that could be done. He had heard of fillings as solid as molten gold, but he never expected to see any; and fortunately such fillings were not necessary. The tooth could be preserved by one much short of solid gold. He had seen, at this meeting, a fine looking filling, which was perfectly preserving the tooth, which had been inserted by a very distinguished operator, and was yet so soft that it could be pierced by a pointed instrument. He said, that while he was up, he would make a remark on the discoloration of crystal gold, and "crystal gold foil." Much had been said in regard to the former changing color; and the charge of impurity had been brought against it. The latter, however, was subject to the same change; and he believed that with both, the fault was not in the material, but in the manipulation. The usual instructions for filling with crystal gold, directed us to begin at the bottom of the cavity, and condense toward the sides, manipulating so as to keep the surface of the filling concave. This, he thought, was incorrect practice. When the surface was concave, he said, that when pressure was made on a piece of gold, in adapting itself to the concavity, it is drawn away from the walls of the cavity. The result is, the filling does not fit tightly to the walls of the cavity, foreign agents are admitted, and discoloration ensues. His mode was to have the filling convex as he progresses,—to keep the plug the fullest in the centre. By this mode, he maintained that the tendency of the gold, under pressure, would be toward the walls of the cavity. And even when the pressure was directly on the centre, it would be like pressure on the keystone of an arch, tending to force the extremes of the arch farther from each other, and consequently, more firmly against the walls.

Dr. STONE selected, by request, a posterior approximal cavity in a lower bicuspid. He would make a V shaped separation, leaving a shoulder to prevent the teeth closing together. He would leave no sharp edges to the filed surface. Having prepared his cavity, he would wedge away the gum with a piece of wood between the necks of the teeth, then dry the cavity with "boiled cotton," and introduce the gold. The part of the cavity next the neck of the tooth, and that next the indentation, are the points to be most closely watched. He uses, for this cavity, pluggers bent to about forty-five degrees, and would use about three of them, one being flat to condense next the gum after the gold is introduced. He uses blocks, or pellets, prepared from loosely folded strips, begins at the bottom of the cavity—the wall next the gum—and finishes near the middle, piercing the plug, or even drilling into it, to introduce the last piece. He would then file off the surface and condense farther, file again, and again condense, and then finish as usual. In reply to a question he said he did not believe it was necessary to have all the moisture out of the cavity.

THIRD DAY—AFTERNOON SESSION.

"*Filling Teeth*," continued.

Dr. PERINE described his manipulations in filling a right superior bicuspid, the pulp dead and the tooth badly broken down. He endeavored to restore the shape of the tooth, and succeeded by preparing the canal, inserting a gold wire into it, leaving it to project, so as to strengthen and retain the filling, as it was built out from the remaining wall. He spent the entire day on the tooth. He said it was difficult to control the saliva in prolonged operations, and remarked that he sometimes gave the patient a saucer, telling them to use it in case of overflow. The result was that there was seldom any overflow. The anxiety being lessened, the salivary glands did not act so freely.

Dr. ROGERS of Utica, described and recommended an instrument for condensing plugs, after all the gold is introduced. He called it a "serrated burnisher," and said it was made by polishing a serrated or grooved instrument with a brush, and its advantage was, that it condenses the gold without cutting it. The pressure is applied with a kind of "rolling motion." He also suggested the propriety of filling deep cavities partly full of some *non-metallic* substance, before using the gold. It was necessary, he said, to study economy with some patients, and if some indestructible, non-metallic substance could be used, without risk to the tooth, an important point was gained. He had tried two metals in the same tooth, supposing that, if the moisture of the mouth were excluded, no galvanic action would ensue, but he found he was mistaken.

Dr. STONE said, that Dr. Talbert of Lexington, had experimented in this method, and was successful thus far.

Dr. DUNHAM thought, that many teeth might be saved by filling deep cavities partly full of some soft material. He reported a case in which osteo-dentine was formed under gutta percha. In small towns especially, some cheap mode is very desirable, if effectual. He thought plaster of Paris a convenient and valuable material to place in the bottom of a deep cavity for this purpose.

Dr. BRANCH said, he uses plaster mixed with slaked lime made from marble, for the bottom of the cavity, and fills over it with gold. He uses the mixture to preserve the color of the tooth, and as a non-conductor.

Dr. TUTTLE said, that in filling the nerve cavity of a front tooth, he would drill through its lingual surface, and fill with the same instruments he would use for a small cavity. He uses serrated points, deep cut, if the instrument be large; not so deep if small.

(At this point, Mr. Mussey, of the Cincinnati *Gazette*, by request of Dr. Bonsall, announced to the Convention, that the *Niagara was in Trinity Bay with her end of the Atlantic Cable, and that she was in perfect connection with the Agamemnon.* As one man, the members arose to their feet and gave spontaneously, three hearty, vociferous cheers. Mr. M. then announced that the exact locality of the Agamemnon was not yet known. A member shouted: "Three cheers for our side, any how!" These were given with a will; and, with happy countenances, the members quieted down to business.)

Dr. WRIGHT said, in regard to the use of plaster in the bottom of deep cavities, it should be known that the plaster can be rendered no harder by the addition of other materials to it. If rendered anhydrous by calcination, he said it would be harder.

Dr. BERRY thought if any experiments were tried in this direction, the French plaster should be used, as it is much harder than that in common use.

Dr. TAYLOR said, good fillings can be made in various ways. If he found an operator making good fillings by any one mode of manipulating, he was not anxious to have him change it for another. By request, he described his method of filling a posterior approximal cavity in a lower molar. He would cut away the tooth until he could see the entire cavity. He must have perfect room to work. He often cut away one third of the grinding surface of the tooth, so that the tongue could displace any food that might lodge in the space. He would form the base of the cavity—the wall next the gum—into an inclined plane, then he would cut under the corners, at the upper portion of the cavity, to form retaining points. He would prepare his gold in blocks, or cylinders, using gold sufficiently adhesive to prevent its unrolling. The first block he would have large enough to extend across the entire base, and a little outside of the cavity. Having prepared his blocks of various shapes and sizes, with some formed into cones for finishing with, he would take the first block, and with a flat instrument, press it firmly against the base of the cavity, and, if large enough, it would stay there. Then he would add block after block, selecting shorter ones as he approached the upper portion of the cavity, condensing each block thoroughly as he put it in. If well condensed, they need not project much beyond the cavity. He had nothing peculiar in his mode of finishing. Being requested to make some remarks on filling the fangs of the upper molars, he said he wanted every part of the nerve removed: and his method of obtaining this, was, to drill through the centre of the crown, and freely expose the pulp cavity, and then take out the nerve with broaches. He thought young operators often failed by not freely opening the cavity. They were too fearful of cutting away the tooth.

Dr. LINDSAY said, he was opposed to wedging the teeth to separate them. There was danger of elongation, or protrusion from the socket, resulting from it. He was in the habit of filing freely; and he never leaves a square shoulder. The farther back in the mouth the larger the V should be made in separating.

Twenty minutes being, on motion, assigned to the consideration of local anæsthesia,

Dr. BRANCH said, that in the subject of local anæsthesia by congelation, he was less interested than the profession. (Dr. B. is the patentee of an instrument for congealing the gums for the purpose of extracting teeth without pain.—REP.) He said this application had many advantages. It was harmless. There might be, he said, cases in which it would do harm, but he could think of no condition in which it would be injurious. As a remedial measure, he said its usefulness extended far beyond the extraction of teeth. It relieved sensitive dentine, periostitis, inflammation of the gums, and kindred pathological conditions. He exhibited the instrument, and described minutely the method of using it. He said he would like to hear the result of Dr. Taylor's experience in the use of it.

Dr. Taylor said, his experience had not been uniform. Sometimes it acted pleasantly,—other times not.

Dr. Tefft exhibited a liquid which he used locally to destroy sensibility and prevent the pain of extraction. He moistens two pledgets of cotton with the liquid and applies them to the gums, for a minute or two, over the tooth to be extracted. He said he had been offered fifteen hundred dollars for the formula, which was no patent, and which he now gave freely to the profession. The recipe is as follows:

Take, Tincture of Aconite,
Chloroform, Alcohol, each, an ounce,
Morphine, six grains. Mix.

Dr. Roberts said, he had extracted some two hundred teeth, with the use of the galvano-electric current. The results varied somewhat, but the patients generally claimed that there was less pain.

Dr. Dunn said, that a lady, now in Delaware, O., had her teeth extracted *two years ago*, under the influence of electricity, as directed under the late patent. She was well pleased with the operation, and said there was little or no pain.

Dr. Berry said, that good old brandy was a first-rate anæsthetic. He had seen it used with good success.

VII. *Miscellaneous Business.*

Dr. Wright exhibited a plaster model of an upper denture which he treated for irregularity. It was a complex case, and was successfully treated; but without cuts we can not make it very intelligible to the reader.

Dr. Allport exhibited a set of continuous gum work, on platinum, which had seriously affected the patient's health; producing severe inflammation of the mouth and neighboring parts, which, he maintained, was not caused by mechanical pressure or irritation, as the patient had previously worn silver plates, and now wears gold ones, with impunity. The gum materials were from the same parcels which he used both before and since the occurrence referred to without any bad effects. He wished for light on the subject.

Dr. ROGERS reported a case in which similar results followed the use of a gold plate. He thought the quality of the plate had nothing to do with the difficulty.

Dr. WATT mentioned a case in which a small twenty carat gold plate produced severe nausea and vomiting—the nausea continuing through a period of some months, as regularly as the plate was worn. The patient was a physician, and was resolved to overcome the difficulty by perseverence. He had not heard for a few weeks, how he was succeeding.

Dr. DUNN exhibited specimens of porcelain work according to Loomis' patent, which he thought had decided advantages over all other styles of work. He maintained that it was cleanlier, stronger, more natural in appearance, easier made, and every way more efficient than other styles.

Dr. REHWINKLE described a case of necrosis of the inferior maxilla, which occurred in his practice. He removed the dead bone, and the teeth it contained. These he exhibited to the Convention, and then presented them to the Ohio College. Altogether it was an interesting case.

Dr. CONWAY made some remarks on his dental lamp. This lamp is patented by Dr. C., and is intended to answer all the purposes for which heat is required in the dental laboratory. Dr. C. exhibited its powers and uses, at various times, in the laboratory of the College, during the sitting of the Convention.

Dr. OSMOND described a method of soldering lower sets, which he had not tested on gold plates, but which was very satisfactory with silver. Owing to the low tones of Dr. O., and some confusion where we sat, we are unable to give his method.

Dr. ALLPORT exhibited an appliance, which was successful after other means failed, in retaining a fractured lower maxilla in proper position. In this case the general surgeon recognized the dental surgeon as his professional brother, applied to him, and received just that assistance which he needed. (A full report of this case is about being published in the Chicago *Medical Journal;* and it will be laid before the readers of the Register at the earliest opportunity.—REP.)

Dr. WILLSON reported a case in which the first bicuspid made its appearance through the cheek, near the mental foramen. It was natural in form and appearance—normal in all respects, except position. He found it quite difficult to extract. The patient's jaw had been fractured in early life.

Dr. LUPTON said he put an amalgam filling in a tooth that was badly decayed; and to prevent the amalgam's showing through the enamel, he laid a portion of gold foil against the anterior wall of the cavity, and put the cement in behind it. The patient, he said, complained of a peculiar sensation about the tooth, especially when the tongue touched it, and he wished to know why. A member responded that he had simply erected a "thunder mill" in the tooth.

FOURTH DAY—MORNING SESSION.

Dr. MURRAY read a paper on diseased antrum, and detailed his treatment of a complicated case. (See the original department of the present number of the Register.)

Dr. ALLEN reported a similar case less marked.

Dr. KELSEY reported three cases in his practice, all successfully treated. After opening the cavity through the socket of the second molar, he injected warm water, followed by a "solution of zinc." Otherwise there was nothing peculiar in his treatment. The cases terminated favorably.

Dr. PERINE reported a case of diseased antrum which seriously affected the sight. The molars on each side were filled with amalgam. He removed these and the patient recovered.

Dr. WRIGHT reported a complicated case of diseased antrum, in a scrofulous patient, which terminated fatally. Also a case of irregularity, in which the lower teeth shut outside of the upper ones. He remarked that when contraction of the jaw takes place from premature extraction, the front teeth go backward, not the back ones forward. (A number of cases were reported, and specimens presented, which were highly interesting to the members, but which can not be described in print to give satisfaction.—REP.)

Dr. McCALLA read a paper on lateral pressure of the teeth. (For this paper, see the original departmen of this number.)

Dr. McKELLOPS reported a case of protrusion of the lower jaw, which he was correcting by the use of the "Fox bandage," as described in Harris' Principles and Practice.

On motion, one hour was devoted to the subject of filling teeth.

Dr. PALMER described his method of filling an approximal cavity in a front tooth. He separates so as to fill from the lingual surface of the teeth. He discriminates in regard to the different methods of separating. If the necks of the teeth are narrow, he wedges. He makes the cavity triangular, with the apex downward; and in inserting the filling, he finishes at the apex. He polishes the filed surface with pumice, and then with rotten stone.

Dr. ROGERS described a file, shaped like a knife blade, which cuts by drawing instead of pushing. With this, he said, it was easy to separate posteriorly, without filing the labial surfaces to any extent.

Dr. FULLER said his old method of filling, was, to begin with soft cylinders, prepared according to the dimensions of the cavity, and finish with hard ones. A cylindrical cavity, he thought, was better filled with non-adhesive, than with adhesive gold. When he resorts to the adhesive style, he uses Watt's & Co's "Crystal Gold Foil." He makes a groove, or pits, at about the junction of the dentine with the enamel. If the cavity is large he inserts a large pellet, using smaller ones as he progresses. When he gets to the groove, he forces the gold into it, and welds it to the mass in the bottom of the cavity. He controls the sublingual ducts by placing a roll of napkin beneath the tongue, and the parotid by bibulous paper. He inserts a wedge of wood, or piece of rubber, between the necks of the teeth, to keep the border of the cavity next the gum, dry.

Dr. FORBES, by request, described his method of preparing cavities. For central cavities, in the upper molars and bicuspids, he uses a straight gouge, beveled from the inner side. With this he cuts down the sides of the cavities, as a mechanic would the sides of a mortice with a paring chisel. For similar cavities in the lower teeth, he has his gouge bent at right angles; for approximal cavities, he uses four gouges, bent at right angles, and the edges facing four different ways. For following the seams in the grinding surfaces, he uses a *v* shaped gouge. He stated that files cut at an angle of sixty or seventy degrees would separate the teeth with less pain and jarring—are pleasanter both to patient and operator. He uses "water stone" to sharpen his instruments; oil stones, he said, were a filthy institution.

Miscellaneous business being again in order,

Dr. CHANDLER made some remarks on hemorrhage of the gums, and his treatment of the same. He reported a severe case of hemorrhage, resulting from lancing the gum around an incisor tooth, to relieve pain. The ordinary astringent applications were used without effect; but a free use of ice water to the head and neck worked like magic. It was instantly arrested, and was not renewed. This case occurred in 1840 or 1841; and, since that, he had resorted to the same course in all obstinate cases, and had found it satisfactory. His theory was, that the cold caused contraction of the muscular coat of the arteries, and prevented the accumulation of blood about the head.

Dr. TUTTLE said, that as a compress, in suppressing hemorrhage after extraction, he uses dry sponge, firmly compressed.

Dr. ROGERS of Utica, said, that pressure was much more reliable than styptics.

Dr. WATT said, that the two were easily combined; and in using styptics it was important to make the proper selection. He referred to a case of hemorrhage which occurred in the mouth of a prominent medical teacher. The patient applied pressure and sulphate of copper; and the hemorrhage was arrested only a few minutes at a time. The use of tannin with no more efficient pressure, made, at once, a permanent arrest. The reason of this may be found in the fact, that the salts formed by a union of albumen with the metallic sulphates, nitrates, etc., is soluble in an excess of albumen. Consequently the coagulum would be dissolved, as the flow of blood continued against it. But when the tannic acid is used, the tannate of albumen is formed, which is not soluble in any constituent of the blood.

Dr. OSMOND said, it was important, in obedience to the laws of hydraulic pressure, to apply the styptics and pressure directly on the mouth of the bleeding vessel. By observing this, he would not despair of any case.

Dr. ATKINSON remarked, that two deaths had occurred in Cleveland, from hemorrhage of the gums during dentition. He spoke favorably of tartaric acid as a styptic.

Dr. CHAS. MERRY, of St. Louis, exhibited an improved drill stock, which was greatly admired by all present.

After a vote of thanks to the profession in Cincinnati for the entertainment and carriage excursion, and to the faculty of the Ohio College, for the use of its halls, the Convention adjourned.

---

In this report we have endeavored to retain all the practical and scientific points presented, without much regard to the exact language of the speaker. As in all popular meetings of the profession, much was said that was mere common place, by way of getting an introduction to that which was more rare and important. For instance, a member would rise—a firstrate fellow too, but a little bashful—and, by way of collecting his thoughts, would tell us he had never been at such a place before,—would tell us where and when he began practice, why he turned his attention to the profession, etc. Now, we suppose, no one expected such remarks to be reported; and we have, accordingly, omitted the *preface*, and have endeavored to give the *body of the work*. We have omitted the "applause," "laughter," etc., and also the flashes of wit—we were after the *science*—but the reader may applaud and laugh as often and as heartily as he pleases, and will probably still fall short of the Convention's efforts in this respect. We have not attempted to report the sayings and doings at *the supper*, as they were, as they should be, far more social than scientific. Below, we give the toasts or sentiments offered and responded to.—REP.

THE BANQUET—REGULAR AND VOLUNTEER TOASTS.

*The American Dental Convention.*—Responded to by the President, Dr. Isaiah Forbes, of St. Louis.

*The Medical Profession:* Ever zealous in the cause of science, we welcome this night to our festive boards; and as fellow soldiers in the great battle with disease, we extend them a cordial greeting.—Responded to by Proffessor L. M. Lawson, M. D. of the Medical College of Ohio.

*Our brethren of the East.*—Response by Dr. John Allen, of New York, formerly of Cincinnati.

*Our brethren of the South.*—Responses by Dr. Chase of Georgia, and Dr. Berry of Mississippi.

*Our brethren of the North.*—Response by Dr. Atkinson of Cleveland.

*Our brethren of the West.*—Responded to by Dr. Allport of Chicago.

Volunteer sentiments:

*The land of Dudley:* Though represented by a STONE, it proves to have been wrought into a highly finished Corinthian Capital.—Response by Dr. Stone, of Kentucky.

*The Granite State:* At each successive meeting, may its representation still be FULLER.—Responded to by Dr. Fuller, of New Hampshire, who closed by offering the following:—

*The Faculty of the Ohio College of Dental Surgery:* In the ripe wisdom of a Taylor, the brilliancy and energy of a Taft and a Smith, the solid and substantial acquirements of a Watt, a Richardson and a Chapman—in the untiring devotion of each and all to their arduous profession, let us discover those qualities of mind and heart, which, while we admire, we should deem it our highest duty to imitate.—Response by Dr. Taft.

*The true professional man:* Always sought—never seeking—sustained alone by his own merit; may his reward be equal to his disinterestedness, and may it be our ambition to emulate his example.—Response by Dr. Watt.

*The Press.*—Responded to by J. D. Caldwell of the Cincinnati *Gazette.*

By Dr. Frank Fuller :—

*The Atlantic Telegraph*—uniting, this day, with its electric chain, two mighty nations of the earth—may no message of war ever thrill along its nerves, but may its utterance ever be, "*Peace on earth—good will to man.*"

With the band playing "The Star Spangled Banner," the banquet closed.

